# Impact of Virtual Disaster Collaboration Exercises on Disaster Leadership at Hospitals in Saudi Arabia

**DOI:** 10.1007/s13753-021-00376-0

**Published:** 2021-11-02

**Authors:** Mohammed Ali Salem Sultan, Amir Khorram-Manesh, Eric Carlström, Johan Berlin, Jarle Løwe Sørensen

**Affiliations:** 1 Healthcare Transformation, Model of Care, Regional Health Directorate, Najran, 66255 Saudi Arabia; 2 Institute of Health and Care Sciences, Sahlgrenska Academy, Gothenburg University, 405 30 Gothenburg, Sweden; 3 Institute of Clinical Sciences, Sahlgrenska Academy, Gothenburg University, 405 30 Gothenburg, Sweden; 4 Department of Research and Development, Swedish Armed Forces Centre for Defence Medicine, 426 76 Gothenburg, Sweden; 5 Gothenburg Emergency Medicine Research Group (GEMREG), Sahlgrenska Academy, 413 45 Gothenburg, Sweden; 6 USN School of Business, Campus Vestfold, University of South-Eastern Norway, 3603 Kongsberg, Norway; 7 Department of Social and Behavioural Studies, University West, 461 86 Trolhättan, Sweden

**Keywords:** Collaboration exercises, Disaster education, Emergency management, Healthcare personnel training, Saudi Arabia

## Abstract

This study measured the impact of virtual three-level collaboration (3LC) exercises on participants’ perceived levels of collaboration, learning, and utility (CLU) at hospitals in the southern region of Saudi Arabia. Our 3LC exercise is a tabletop training tool used to facilitate disaster education and document CLU. This model enables the practitioner to acquire new knowledge and promotes active learning. An English version of the CLU scale, the validated Swedish survey tool, was applied to 100 healthcare managers or leaders in various positions at both the operational and tactical levels after conducting the 3LC exercises. The response rate was 100%, although not all questions were answered in some cases. The results show that most participants strongly agreed that the exercises focused on collaboration (*r*^2^ = 0.767) and that they had acquired new knowledge during the exercises. There was a statistically significant association between participation in the collaboration exercises and perceived learning (*r*^2^ = 0.793), as well as between perceived learning and utility (*r*^2^ = 0.811). The collaboration exercises enhance the perceived effects of CLU. They also improve the ability of participants to adapt situational strategies to achieve a safer society. Although exercises were conducted virtually, they were well received by the participants and achieved a value M = 4.4 CLU score, which opens up new dimensions in collaboration simulation exercises.

## Introduction

Increasing numbers of disasters and public health emergencies have resulted in individual and material damages, deaths, and disabilities (Oktari et al. 2020). The majority of these negative outcomes are preventable through enhanced emergency system readiness (Coppola 2006; Carter 2008; Torani et al. 2019). The immediate and appropriate response to such emergencies requires individual and organizational mitigation and preparedness, collaboration and coordinating abilities, communication skills, global awareness, and mutual situational awareness. These elements create bridges spanning multiagency borders and utilize all components of surge capacity, that is, staff, supplies, structures, and systems, to achieve a smooth and adequate transfer and medical management of victims to and within medical facilities (Sagun et al. 2009; Khorram-Manesh 2020). It is evident that actions, structures, and systems implemented within this multiagency approach to disasters and public health emergencies should be harmonized to avoid unnecessary time- and resource-consuming discussions and shortcomings (Boin and ‘t Hart 2010).

An appropriate level of preparedness may be achieved through either exposure to disasters or proper educational programs (WHO 2017). In addition, readiness to respond to an emergency requires theoretical and practical knowledge (Khorram-Manesh et al. 2015). Sultan, Khorram-Manesh et al. (2020) have indicated that healthcare workers who avoid taking part in emergency management often lack the practical element of readiness. In contrast, knowledgeable healthcare staff seems to have more confidence to act and are more willing to participate in unexpected situations.

Exercises can increase the knowledge and confidence of staff while allowing them to practice in an environment without risk of harm to patients (Andersson et al. 2014; Khorram-Manesh, Lupesco et al. 2016). Various types of exercises exist, including real-time, full-scale exercises with high costs and insufficient training in whole chains of action; three-level collaboration (3LC) and tabletop exercises; and more sophisticated simulation training, such as the medical response to major incidents (MRMI) (Khorram-Manesh, Berlin et al. 2016). There are pros and cons to each of these methods; however, training staff should not only provide opportunities for learning and skill development, but should also include training in multiagency collaboration and coordination in an environment that allows for mistakes and correction (Klabbers 1999; Berlin and Carlström 2008a). Such a model enables the transfer of new knowledge into practice and promotes active learning, which can be innovative and applicable to real life (Revell and Wainwright 2009; WHO 2021).

Some of the aforementioned exercises have a more distinct focus on collaboration, interagency participation, and joint decision making—for example, 3LC and MRMI, which are both validated (Charman 2013; Khorram-Manesh, Lupesco et al. 2016). The former requires only simple instruments and a relatively small space, while the latter uses more sophisticated instruments and requires more space. Both models aim to develop synchronous collaboration and strengthen perceived levels of learning and utility by focusing on flexibility, improvisation, and joint evaluations. The primary target of the 3LC model is the collaborative elements in mutual tasks; this model aims to reduce organizational barriers (Khorram-Manesh, Berlin et al. 2016). The MRMI targets individual decision making but also delves deeper into the medical perspectives of the management process (Roud and Gausdal 2019).

Collaborative learning in this study is limited to Stein’s (1997) and Klabber’s (1999) perspectives concerning the learning methods of institutions and, subsequently, the differences between first- and second-order learning. In first-order learning, the participants cannot transfer their new knowledge into practice, either because they are structurally unable or unwilling to do so. In contrast, in second-order learning, the participants acquire new knowledge and apply that knowledge in real-life situations. Collaboration exercises frequently motivate individuals to obtain new skills and professional knowledge from each other (Berlin and Carlström 2011) and support processes that encourage collaboration (Berlin and Carlström 2008a). Participants prefer familiar standardized working patterns, and so participating in collaboration exercises can result in the integration of useful theoretical structures and practices into the work environment (Berlin and Carlström 2008a).

Scientifically verified and well-established exercise models can be used in the context of disaster-prone areas to facilitate disaster education and document collaboration success, learning, and usefulness. The Kingdom of Saudi Arabia (KSA) is a disaster-prone area in need of disaster education (Sultan, Sørensen et al. 2020). In a recent study carried out in the KSA (Al Thobaity et al. 2019), participating doctors and nurses from eight hospitals reported that disaster plans must include five necessary components, namely surge capacity, security, decontamination, communication, and survivor support. These components could strengthen hospitals’ preparedness for disaster response and recovery. In another study (Mani et al. 2020), a systematic scoping review focused on armed conflict areas and included a number of common disaster core competency domains, such as disaster planning, communication, safety, chemical, biological, radiological, nuclear and explosive and personal protective equipment (PPE) that could be enhance by workplace education and drills for hospital and healthcare providers.

Nevertheless, there is often a lack of collaboration in the interagency approach to a disaster and an appropriate educational tool is needed to strengthen interagency and intra-organizational partnership and to optimize preparedness. Such demands might be met by 3LC and MRMI simulation exercises, which facilitate follow-up and development evaluation. In particular, 3LC, which has more precise requirements and focuses on organizational attitudes, may be implemented in both central and remote areas of the country.

The current COVID-19 pandemic has been associated with many changes in global perspectives and especially in the development of new digital initiatives (Budd et al. 2020). One such area of development has been virtual learning and education, which has seen rapid growth and popularity as people’s attitudes towards technology and ease of access have shifted, making it easier for students to continue their education in different and difficult circumstances (Spiceland and Hawkins 2002; Sahu 2020). This massive unplanned shift from traditional learning to online learning has changed the methods used by medical institutions to offer their courses to students (Schwartzstein and Roberts 2017). Our study attempts to measure the impact of virtual 3LC course exercises on participants’ perceived levels of collaboration, learning, and utility (CLU) in a hospital context.

## Materials and Methods

This study employed a quantitative research method that employs a survey design to assess the intervention of a formalized exercise model promoting the learning and usefulness of three-level collaboration (3LC) in a Saudi Ministry of Health (MOH) hospital context.

### Instrument

This study applied an English version of the Collaboration, Learning, and Utility (CLU) Scale. The CLU-Scale is a validated Swedish survey tool specially designed to measure exercise participants’ perceived levels of collaboration, learning, and utility. A CLU survey was developed in different stages by experts in the accidents and disasters field. It was formulated on theories that distinguish between sequential, parallel, and synchronous collaboration (Berlin and Carlström 2008b); learning theories derived from Stein and Klabbers’ perspectives on how institutions learn; and the differences in learning orders between the first and second learning orders (Stein 1997; Klabbers 1999). A comparison can be made between collaboration exercises as similar studies have applied the CLU tool (Berlin and Carlström 2015; Sørensen et al. 2018, 2020). Our survey was comprised of 22 items distributed between two sections: a demographic section consisting of 5 items and an evaluation section composed of 17 items across three dimensions—collaboration (C), learning (L), and utility (U)—displayed in Table 1. The C dimension evaluated the perceived collaboration characteristics, the L dimension confirmed collaboration-related lessons, and the U dimension addressed the transfer of value to real-life scenarios. The CLU scale captured the participants’ perceptions based on a 5-point Likert scale ranging from 1 (strongly disagree) to 5 (strongly agree).

**Table 1:** The collaboration, learning, and utility scale (CLU-scale)

Dimensions	Items
C	The exercises were focused on collaboration
C	Sufficient forms of discussion were provided
C	There were opportunities to improvise
C	Personnel in need of exercise participated
C	I performed well-known activities
C	Collaboration was initiated immediately
C	Clear instructions for collaboration were presented
C	My points of view were regarded
L	I learned new things during the exercise
L	I learned about others’ organizational aspects
L	I learned about others’ communication patterns
L	I learned about others’ prioritizing of activities
L	I learned others’ concepts and abbreviations
U	Based on what I learned, the exercises were useful to real-life activities
U	Based on what I learned, the exercises were useful to command officers
U	Based on what I learned, the exercises were useful to ordinary operative staff
U	Based on what I learned, the experiences from the exercises impact my daily work

Dimensions: *C* collaboration, *L* learning, *U* usefulness

### Setting

The study data were collected after conducting the 3LC exercises over three days in October and November 2020, from 10 MOH hospitals, at the Simulation and Skills Development Centre of the Regional Health Directorate in Najran, KSA. Najran region is located in the southern part of the KSA, which is exposed to the potential risk of disasters such as sandstorm, flash floods in wadis, building collapse, and armed conflict at the border.

### Population and Sample

The study participants were healthcare leaders working at 10 MOH hospitals in the Najran region, KSA. Participants included staff in various positions at both the operational and tactical levels, namely, hospital director, medical director, nursing director, emergency and disaster director, public health director, financial director, and support services director. The sample size was set at *n =* 100 healthcare managers/leaders based on the power calculation (Raosoft 2004), assuming 4.5% precision with 50% prevalence and in total population size of 124 with a 95% confidence interval specified limits.

### Data Collection and Procedures

Due to the COVID-19 pandemic, which forced most countries’ airports to shut down, lengthy meetings were held through virtual communication between the experts from Sweden and Norway as well as health practitioners with experience in disaster management from Saudi Arabia, in order to teach, train, and prepare them on the course scenarios. In addition, the first course was held through virtual communication between experts and participants, with five qualified health care leaders receiving training from the experts in order to provide leadership on the ground and conduct the remainder of the 3LC course.

**Fig. 1:**
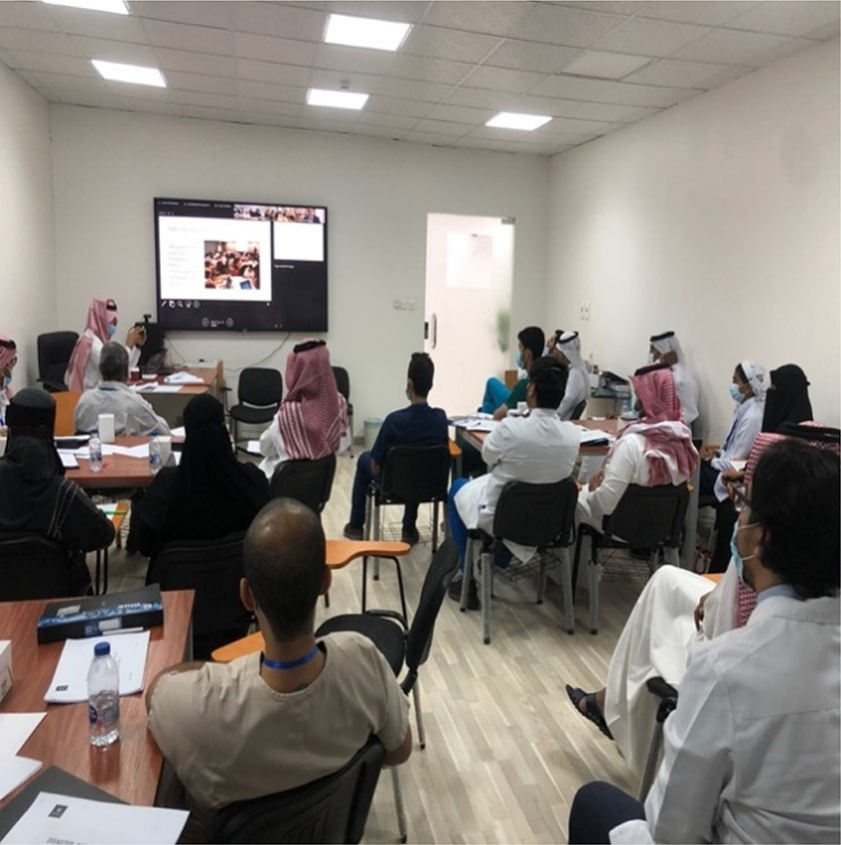
Theoretical lectures from the Saudi side. Photograph by M. Sultan, Najran, 13 October 2020

**Fig. 2:**
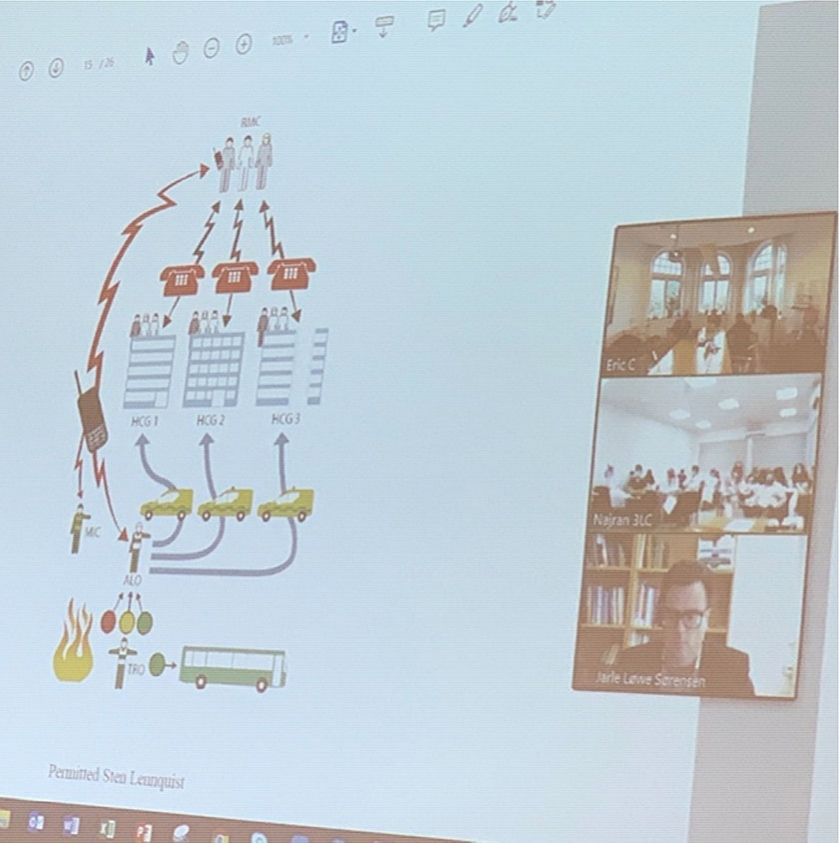
Theoretical lectures from the Swedish side. Photograph by J. Berlin, Gothenburg, 13 October 2020

The theoretical lectures were presented to those enrolled in the 3LC exercise courses (Figs. 1, 2). Afterward, the participants were divided among four tables, with a leader, a physician, a nurse, and a support services worker at each table. Participants received the necessary instructions to engage in side-by-side tabletop exercise work in two simulated reality. The two case scenarios are:

1. Scenario one (critical care evacuation within a hospital): a healthcare team is working at a Critical Care Unit and has 10 patients, 30−50 years old. There are three categories: 3 respiratory cases, parenteral nutrition, extensive injuries; 3 cases of severe injuries, intravenous infusion and receiving inotropic medication (they are awake and have spontaneous breathing); and 4 cases have newly undergone surgery due to trauma or heart disease (they are awake, have spontaneous breathing and are mobilized to wheelchair). The event: an alert from hospital management that explosives are located at the main building of the hospital and the critical care unit is threatened. It is impossible to move or disarm the explosives within the next 2−3 hours. There is an immediate need to evacuate patients and staff to clear hospital buildings nearby. Elevators are impossible to use because of suspected triggers and explosives. Support from external staff is not expected. Patients need to be moved 5−6 floors down by the stairs. Evacuation has to start as soon as possible. The healthcare team must report to the hospital management within 15 minutes on strategy, priorities, risk analysis, and possible losses.
2. Scenario two (mass casualty scenario): a healthcare team is sent from the hospital to a disaster site in the vicinity of the hospital. The event: a post-Ramadan festival is going on, and there are reports of shooting in the area from automatic weapons directed at the crowd. Before your team leaves the hospital, you are informed of casualties—around 100 people are dead and wounded. More teams are prepared, but your team is first in line. The police department and the military have secured the area and captured terrorists. About 10 ambulances arrive at the same time as you arrive at the site. The area, a park in the town, is roped off from the public by the police. There are about 75 wounded and dead, all in lying or sitting position. How do you act? You are supposed to give a report to the hospital management as soon as possible.

**Fig. 3:**
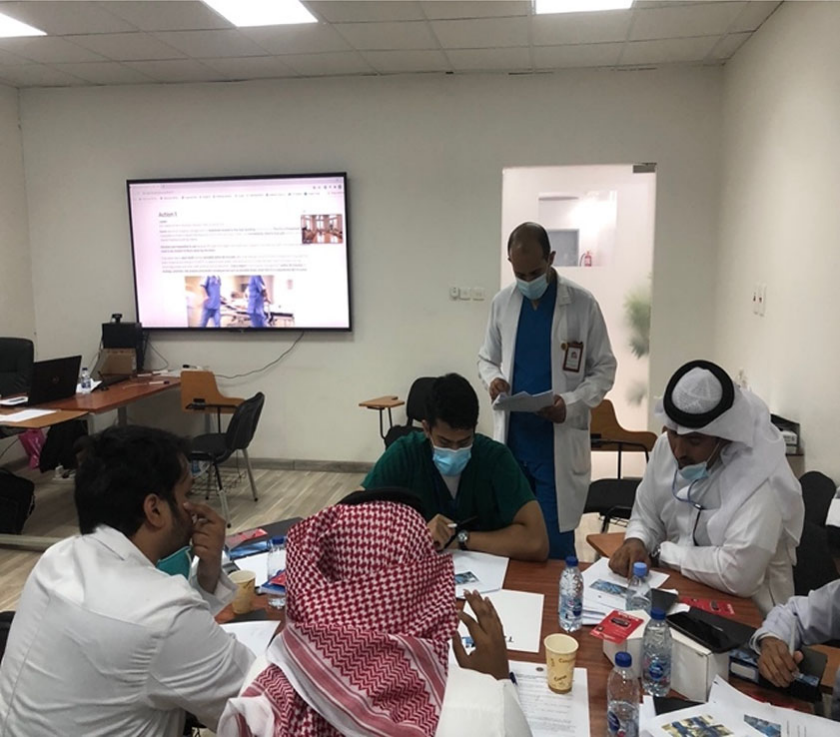
Exercise leaders count the time while the participants are working on scenarios. Photograph by M. Sultan, Najran, 13 October 2020

**Fig. 4:**
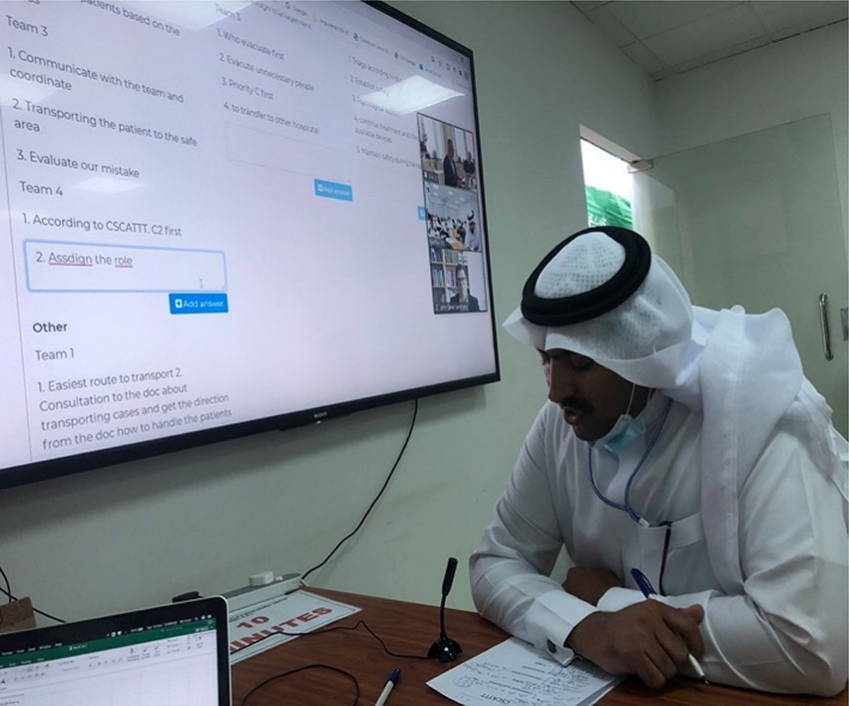
One of the participants (a team leader) presents the Swedish-Norwegian experts his team skills in handling the event. Photograph by M. Sultan, Najran, 13 October 2020

During each scenario exercise, the exercise leaders measured the time (Fig. 3) elapsed from the presentation of the scenario. After completing the specified allowed time for working on the scenario, each team showed how it individually handled the event (Fig. 4). The exercise leaders then provided the participants with a quick review of the scenario. After that, participants at each table were asked to identify what they could have done differently.

At the end of the course, online CLU survey instruments were sent separately to each participant’s email (*n =* 100; online form) hosted on the 3LC platform.

### Data Analysis

The homogeneity of the items in the subscales of the CLU survey was analyzed by calculating Cronbach’s alpha using the Statistical Package for the Social Sciences (SPSS) version 20. Cronbach’s alpha was 0.987, which shows high internal consistency and, according to Brace et al. (2006), is considered satisfactory. The authors used regression analysis to identify independent variables significantly associated with the dependent variable. All significant variables found in bivariate regression analysis were included in the multivariate regression analysis.

### Ethics

Informed consent was obtained from all study participants. They were informed regarding their voluntary participation and the possibility of withdrawing from the study whenever they choose to, with no consequences. Collected data were stored at the research center in each hospital. The information provided was subject only to research purposes, all data were handled confidentially, and the researchers could not disclose the respondents’ identities at any time, no matter the circumstance. An ethical committee certificate of approval for the study was obtained from the Institutional Review Board at the Regional Health Directorate in the Najran region (IRB Log Number 2021-19 E; date of approval: 7 April 2021).

## Results

The results have shown various proportions according to the collaboration, learning, and utility dimensions of the used scale. Bivariate and multivariate regressions were used to identify the associated independent and dependent variables.

### Demographics

One hundred participants took part in the survey (*n* = 100), of which 37% were females, and 63% were males. The overall response rate was 100%, that is, all participants replied to the survey. However, some of the participants did not answer all questions. Consequently, questions with missing answers were categorized as a negative response (Total disagreement), and included in the results. Respondents younger than 23 and older than 59 were the least represented. Only 5% of participants were below age 25, and 5% were older than 50. The mean age group was 29–38 and was the age group that was most represented by the respondents (52%). About 37% of the respondents were in the nursing department. Experience ranged from 5 years and less to more than 25 years. Only 4% of the staff had more than 25 years of experience. Most workers had 5–9 years of experience (30%).

### Collaboration

A total of 61% of the participants strongly agreed that the exercise focused on collaboration, while 24% mildly agreed. Only 7% disagreed that the exercise focused on collaboration, with the mean M = 4.34, and standard deviation SD = 1.056. About 88% either strongly or mildly agreed that acceptable forms of discussion were provided in the exercise. Only 2% mildly disagreed with the statement, and 5% strongly disagreed (M = 4.44, SD = 1.038). Whether there were opportunities to improvise, 63% strongly agreed, and 21% mildly agreed. About 8% agreed and 7% either mildly or strongly disagreed (M = 4.33, SD = 1.120). The majority of participants strongly agreed that personnel in need of the exercise participated (55%), while 27% mildly agreed. About 6% strongly disagreed that personnel who needed it participated in the exercise (M = 4.31, SD = 1.124). In total, 59% of the participants strongly believed that they performed well-known activities, while 27% mildly agreed. Only 8% disagreed (M = 4.31, SD = 1.089). In total, 91% agreed that collaboration was initiated immediately, while only 7% strongly disagreed (M = 4.29, SD = 1.192). When asked whether clear instructions on collaboration were presented, 64% strongly agreed, while 22% mildly agreed. Only 10% disagreed (M = 4.38, SD = 1.052). Half of the participants strongly agreed that their points of view were regarded, while only 8% disagreed (M = 4.21, SD = 1.052). The overall mean of collaboration was 4.317, while SD was 0.954.

### Learning

Most of the respondents either strongly (71.0%) or mildly (19%) agreed that they had learned new things during the exercise (M = 4.48, SD = 1.049). If the exercise participants felt that they had learned about others’ organizational aspects, 2% mildly disagreed while 6% strongly disagreed. A total of 87% either strongly (68%) or mildly (19%) agreed to the statement (M = 4.38, SD = 1.170). In total, 75% either strongly or mildly agreed that they had learned how others prioritize their activities (M = 4.31, SD =1.178), while more than two-thirds (86%) had learned about the communication patterns of others. Here, 7% remained neutral (M = 4.40, SD = 1.082). Eighty-six out of 100 participants agreed, while six participants disagreed to learning new concepts and abbreviations (M = 4.36, SD = 1.142). The general mean for learning was 4.406, while the SD summed up to 1.031.

### Utility

Most of the participants (88%) found the exercise useful for real-life activities. Only 7% disagreed that the exercise was useful (M = 4.41, SD = 1.083). While 8% disagreed and 3% remained neutral that the exercise was useful for commanding officers, 88% agreed to it (M = 4.34, SD =1.157). More than half of the population (63%) strongly agreed that the exercises were useful for ordinary operative staff. Here, only 5% remained neutral (M = 4.39, SD = 1.034). Finally, 88% agreed that the exercise experience would affect their daily work, while 4% remained neutral, and 8% disagreed (M = 4.39, SD = 1.081). The overall mean for the utility was 4.399, while the standard deviation was 1.032.

Overall means and standard deviations of collaboration, learning, and usefulness are outlined in Table 2.

**Table 2:** Mean values of collaboration, learning, and utility (CLU) dimensions

	Mean values	Standard deviation
Collaboration	4.317	0.954
Learning	4.406	1.031
Utility	4.399	1.032

### Bivariate Analysis

The authors used the bivariate analysis to determine the relationship between the variables of collaboration exercises and learning, and between learning and usefulness.

#### The Relationship between Collaboration Exercises and Learning

Bivariate analysis (Table 3): The significant relationship was between learning and the item “The exercises were focused on collaboration” (*r* = 0.877, *r*^2^ = 0.767, F = 316.307, p ≤ 0.000), while “Sufficient forms of discussion were provided” had a *r*-value of 0.752 (*r*^2^ = 0.561, F = 123.641, p ≤ 0.000).

**Table 3:** Bivariate regression of the collaboration dimensions of learning

Dependent variable: learning Independent variables: collaboration characteristics of exercises				
	Pearson’s *r*	R-Square	F-Value	Significance (p)
The exercises were focused on collaboration	0.877	0.767	316.307	0.000
Sufficient forms of discussion were provided	0.752	0.561	123.641	0.000
There were opportunities to improvise.	0.776	0.598	142.334	0.000
Personnel in need of exercise participated	0.738	0.540	113.869	0.000
I performed well-known activities	0.745	0.550	118.148	0.000
Collaboration was initiated immediately	0.805	0.645	173.537	0.000
Clear instructions on collaboration were presented	0.890	0.790	361.927	0.000
My point of view was regarded	0.717	0.509	100.440	0.000

The item “There were opportunities to improvise” had significance levels *r* = 0.776 (*r*^2^ = 0.598, F = 142.334, p ≤ 0.000), and “Personnel in need of exercise participated” followed closely with a significance level of *r* = 0.738 (*r*^2^ = 0.540, F = 113.869, p ≤ 0.000. The item “I performed well-known activities” had a *r-*value of 0.745 (*r*^2^ = 0.550, F = 118.148, p ≤ 0.000), and “Collaboration was initiated immediately” had a *r*-value of 0.805 (*r*^2^ = 0.645, F = 173.537, p ≤ 0.000).

The item “Clear instructions on collaboration were presented” had the strongest relationship with learning, with a *r*-value of 0.890 (*r*^2^ = 0.790, F = 361.927, p ≤ 0.000), and the item “My points of view were regarded” had a *r*-value of 0.717 (*r*^2^ = 0.509, F = 100.440, p ≤ 0.000).

The multivariate analysis results (Table 4) show that the relationship between the collaboration dimensions of learning and the variables “The exercises were focused on collaboration” and “Clear instructions about collaboration were presented” were significant. The collaborative characteristics predicted 79.3% (*r*^2^^*=*^ 0.793) of the learning variance, meaning that the remaining 20.7% of the predicted variance was unaccounted. The regression analysis indicated an 89.2% (*r* = 0.892) covariance between learning and collaboration characteristics. According to Cohen (2013), this is a sufficiently strong covariation.

**Table 4:** Multiple regression of the collaboration dimensions of learning

Dependent variable: learning Independent variables: collaboration characteristics of exercises			
	Bivariate regression standard beta	Multivariate regression standard beta	Significance (p)
The exercises were focused on collaboration	0.877	0.341	0.002
Sufficient forms of discussion were provided	0.752	−0.009	0.919
There were opportunities to improvise	0.776	0.019	0.824
Personnel in need of exercise participated	0.738	0.016	0.841
I performed well-known activities	0.745	0.000	0.996
Collaboration was initiated immediately	0.805	0.120	0.201
Clear instructions on collaboration were presented	0.890	0.418	0.000
My point of view was regarded	0.717	0.083	0.314

*r* = 0.892, *r*^2^ = 0.793, Significance = p < 0.05

#### The Relationship between Learning and Usefulness

The bivariate regression results (Table 5) show that the most profound significance was between usefulness and the item “I learned new things during the exercise” (*r* = 0.878, *r*^2^ = 0.769, F = 326.812, p ≤ 0.000), followed by “I learned others’ concepts and abbreviations” (*r* = 0.875, *r*^2^ = 0.764, F = 314.478, p ≤ 0.000), and “I learned about others’ organizational aspects” (*r* = 0.858, *r*^2^ = 0.733, F = 266.872, p ≤ 0.000). The item “I learned about others’ communication patterns” received a *r*-value of 0.858 (*r*^2^ = 0.733, F = 270.429, p ≤ 0.000). The last item was “I learned about others’ prioritization of activities” with a *r*-value of 0.828 (*r*^2^ = 0.764, F = 314.478, p ≤ 0.000).

**Table 5:** Bivariate regression of the learning dimensions of usefulness

Dependent variable: utility Independent variables: learning characteristics of exercises				
	Pearson’s *r*	R-Square	F-Value	Significance (p)
I learned new things during the exercise	0.878	0.769	326.812	0.000
I learned about others’ organizational aspects	0.858	0.733	266.872	0.000
I learned about others’ communication patterns	0.858	0.733	270.429	0.000
I learned about others’ prioritization of activities	0.828	0.683	209.812|	0.000
I learned others’ concepts and abbreviations	0.875	0.764	314.478	0.000

Multivariate analysis (Table 6): The perceived learning items predicted 81.1% (*r*^2^ = 0.811) of the usefulness variance, meaning that the remaining 18.9% of the predicted variance was unaccounted. The items that were found to be significant were “I learned new things during the exercise” (p = 0.003) and “I learned others’ concepts and abbreviations” (p = 0.000). These results (*r* = 91.4%) indicate a strong relationship between learning and usefulness (Cohen 2013).

**Table 6:** Multiple regression of the learning dimensions of usefulness

Dependent variable: utility Independent variables: learning characteristics of exercises			
	Bivariate regression Standard beta	Multivariate regression standard beta	Significance (p)
I learned new things during the exercise	0.878	0.333	.003
I learned about others’ organizational aspects	0.858	0.239	.140
I learned about others’ communication patterns	0.859	−0.006	.970
I learned about others’ prioritization of activities	0.828	−0.041	.730
I learned others’ concepts and abbreviations	0.875	0.432	.000

*r* = 0.914, *r*^2^ = 0.811, Significance = p < 0.05

## Discussion

In this study, 3LC exercises were conducted for the first time through virtual communication between experts from Sweden and Norway and health practitioners and participants from Saudi Arabia. Although online learning has long been recognized as a practical learning tool (Aronoff et al. 2010), its use can be problematic for students who are used to student-teacher interaction in a classroom environment and may impact student participation and the expression of their opinions (Middleton 1997; Al-Fahad 2009). However, a study by Elfaki et al. (2019) compared the perceptions of a group of nursing students about virtual learning versus traditional education and found that students had a positive attitude towards online education, indicating it is a viable learning method. A recent study indicated the importance of virtual education, highlighting that most health and medical colleges and simulation-based healthcare have developed virtual learning through their platforms and urged educational establishments to do likewise (Tabatabai 2020). Participants were equipped with visual and audio virtual communication tools during this study, and five practitioners with experience in disaster management were trained beforehand in leading the exercise.

A study (Brinjee et al. 2021) conducted in the KSA stated that the nurses with less experience in emergency departments (EDs) had significantly lower levels of disaster management knowledge than those who had spent long periods working in EDs in terms of understanding the incident management systems and its components. Consequently, these authors highly recommended that there must be a disasters course included in nursing curriculums, intensive training courses, mock drills, and simulations (Brinjee et al. 2021). The outcomes of these first virtual 3LC exercises show that the majority of participants strongly agreed that the exercises focused on collaboration, that participants had learned and acquired new knowledge during the exercises, and that the exercises were helpful for real-life activities. There was a statistically significant association between the exercises, collaboration, and learning. The collaboration exercises in this study could be regarded as having improved learning, since the CLU dimensions had a value M = 4.4, making the overall effect of the 3LC course satisfactory.

These results may suggest that people perceive collaboration to be essential in day-to-day activities and in solving emerging issues in the workplace (Perry 2004; Magnussen et al. 2018; Sørensen et al. 2018). A relatively high percentage of participants agreed that the exercises focused on collaboration and the people who attended needed the exercises in their daily activities, 85 and 82%, respectively. This supports the assumption that exercises motivate people to address interorganizational challenges during significant incidents (Fattah et al. 2012). In addition, collaboration exercises will contribute to providing opportunities to tackle issues in individual organizations that are unable to handle these challenges on their own and need additional personnel collaboration support to help management deal with the adverse outcomes of a crisis (Huxham and Vangen 2005; Berlin and Carlström 2011; Powley and Nissen 2012).

Not engaging sufficient collaboration in times of crisis may affect society’s ability to deal with adverse consequences (Sawalha 2014), making it harder for strategic leaders to impose order and meet social expectations (Boin and Bynander 2015). A lack of collaboration may further result in less resilience, flexibility, and efficiency in dealing with disaster situations (Jung and Song 2015). Powley and Nissen (2012) confirmed that collaboration exercises contribute to helping managers and societies to deal successfully with the adverse effects of a crisis. The present findings indicate that clear instructions and ample opportunities for discussion during and after an exercise are important in order to acquire learning. The present study shows that improvising, jointly evaluating, and testing new and alternative strategies among different sectors promote success in the management of emergencies. A major crisis may necessitate a greater emphasis on flexibility, for example, sequential and parallel collaboration. Alexander (2000) argues that, since exercises alone cannot adequately promote emergency management learning, engaging with scenarios bridges the gap between classroom training and practice during actual disasters. Our study’s results suggest that collaboration exercises do not depend as much on the task itself, or the elements involved in the exercises, as they do on working together. The essence of the exercises is that each individual with their own professional knowledge is faced with situations and tasks in which they have to adapt and work together with colleagues who have different professional skills and views (Scholtens 2008).

Gredler (1992) reported that learning might be improved if the personnel is given time for discussion and reflection. Only half of the respondents in the present study strongly felt that their views were regarded. Space to allow participants to air their views and consider the network approaches to understanding the significance of bilateral trust between agencies and authorities is therefore essential (Kapucu and Garayev 2011). Exercises are more effective when the practical sessions are held in tandem with open forums and discussions by the participants to incorporate the day-to-day experience of participants into the theoretical education curriculum (Moynihan 2008). Regression analysis showed marginally stronger covariation between the perceived learning and usefulness dimensions, than between the collaboration and learning dimensions of the exercises. This suggests that participants related their learning to real-life events (Drennan and McConnell 2007).

This study emphasizes the usefulness of virtual exercises and the importance of collaboration exercises. Working together in crisis management enhances the perceived utility of exercises in real accident work, develops the ability to change strategies based on the existing situation, and strengthens attempts to make a safer society.

## Limitations

Even though our results were largely significant, the study was limited in terms of the scope of data collected. The data were only collected from a small number of participants. This constraint reduces the odds of reaching a reliable conclusion or determining results from which suitable forecasts can be made (Coughlan et al. 2009). According to Eisenhardt and Graebner (2007), this can be mitigated through qualitative methods such as observations and interviews to limit informant bias and increase comprehensive insight. Receiving relatively invalid responses was possible because the meanings of collaboration, learning, and utility may have been ill-defined or misunderstood by the participants. The 3LC course was conducted in Saudi Arabia for the first time and in only one of 13 administrative regions (Najran) of the KSA, which is by no means the most important or populous province. Thus it is not possible to generalize the results of this study to all state agencies and regional societies.

## Conclusion

Previous studies have reported a lack of formal educational resources among KSA nurses and have emphasized the need to engage medical personnel in training courses and simulation exercises on the mitigation, preparedness, and response core competencies of disaster management (Al Thobaity et al. 2016). Such requirements might be more difficult to meet in a pandemic. This study confirms the feasibility of three level collaboration exercises conducted virtually. Our work also demonstrates that learning depends on collaboration practices and that collaboration exercises before crises can help to build qualities that people can apply in daily life. Collaboration elements exercised in this study contributed to perceived learning. There was a strong covariation between participation in the participants’ collaboration exercises and perceived learning and utility. The virtual three level collaboration exercises were well-received by the participants and achieved an acceptable collaboration, learning, and utility score. The results of this study open up the possibility of remote education in disaster management, at least from an organizational perspective, in a world with an increasing number of disasters and public health emergencies (Khorram-Manesh and Burkle 2020).
